# Supplementary material for: Autism spectrum disorder-like behavior caused by reduced excitatory synaptic transmission in pyramidal neurons of mouse prefrontal cortex
Source: Nat Commun. 2020 Oct 12;11:5140. doi: 10.1038/s41467-020-18861-3 (PMC7552417; doi:10.1038/s41467-020-18861-3)
Supplement: Supplementary file 1 — Supplementary Information [file 41467_2020_18861_MOESM1_ESM.pdf]

**Supplementary information**

# Autism spectrum disorder-like behavior caused by reduced excitatory synaptic transmission in pyramidal neurons of mouse prefrontal cortex

**Sacai et al.**

# Supplementary Figures

## Supplementary Fig. 1

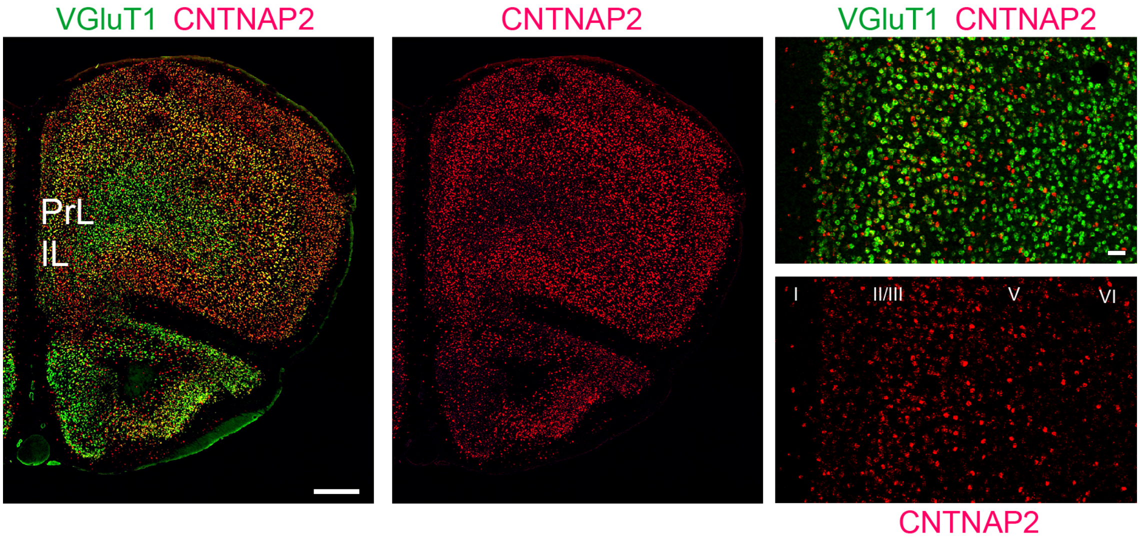


**Supplementary Fig. 1 | Expression of CNTNAP2 in the adult mouse PFC.**

Expression of mRNAs for CNTNAP2 in the adult mouse PFC. Images from double fluorescence in situ hybridization analyses to detect mRNA for CNTNAP2 (red) and VGluT1 (green) in layer I, II/III, V and VI of the PFC. Scale bars, 500 μm (left), 50 μm (right). PrL: pre-limbic, IL: infra-limbic.

**Supplementary Fig. 2**

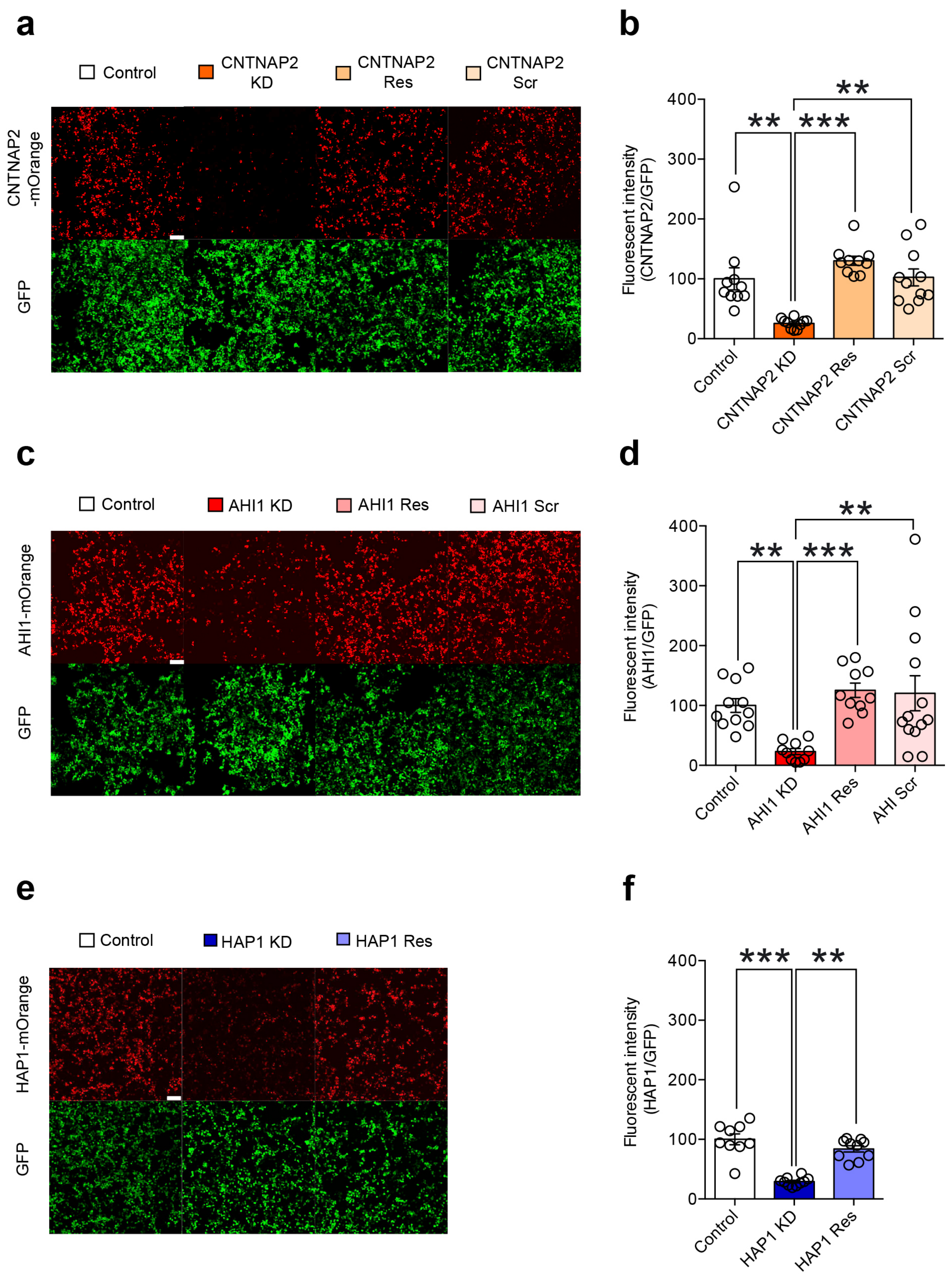

**Supplementary Fig. 2 | Specificity and efficacy of the microRNA vectors for CNTNAP2 knockdown, AHI1 knockdown, HAP1 knockdown, CNTNAP2 rescue, AHI1 rescue, and HAP1 rescue.**

**a, c, e,** Representative images for mOrange (red) and EGFP (green) in HEK 293T cells. An mOrange-fused cDNA vector (for the expression of CNTNAP2 (a), AHI1 (c), or HAP1 (e)) was transfected with an EGFP expression vector (the 1st columns from the left; Control), with the EGFP expression vector containing a microRNA against each gene (the 2nd columns from the left; a, CNTNAP2 KD; c, AHI1 KD; e, HAP1 KD; for showing the effect of knockdown of each gene), or the EGFP expression vector containing a scramble microRNA (see Methods) against each gene (the 4th columns from the left; a, CNTNAP2 Scr; c, AHI1 Scr; for a negative control). An mOrange-fused RNA interference (RNAi) resistant cDNA vector (see Methods) for the expression of CNTNAP2 (a), AHI1 (c) or HAP1 (e) was transfected with the EGFP expression vector containing the microRNA against each corresponding gene (the 3rd columns from the left; a, CNTNAP2 Res; c, AHI1 Res; e, HAP1 Res; for showing the specificity of the microRNA against each gene). Scale bar, 100 μm. **b,** Summary bar graphs showing the fluorescence intensity of mOrange relative to that of EGFP from control (white column, n = 10 images from 2 wells), CNTNAP2-KD (deep orange column, n = 10 images from 2 wells), CNTNAP2-Res (orange column, n = 10 images from 2 wells), and CNTNAP2-Scr (light orange column, n = 11 images from 2 wells) cells. **d,** Similar to b, but the results for AHI1, from control (white column, n = 11 images from 2 wells), AHI1-KD (deep red column, n = 10 images from 2 wells), AHI1-Res (red column, n = 10 images from 2 wells), and AHI1-Scr (light red column, n = 13 images from 2 wells) cells. **f,** Similar to b and d, but the results for HAP1 from control (white column, n = 9 images from 2 wells), HAP1-KD (deep blue column, n = 10 images from 2 wells), and HAP1-Res (blue column, n = 10 images from 2 wells) cells. $**P < 0.01$, $***P < 0.001$ (Dunn test). Source data are provided as a Source Data file.

**Supplementary Fig. 3**

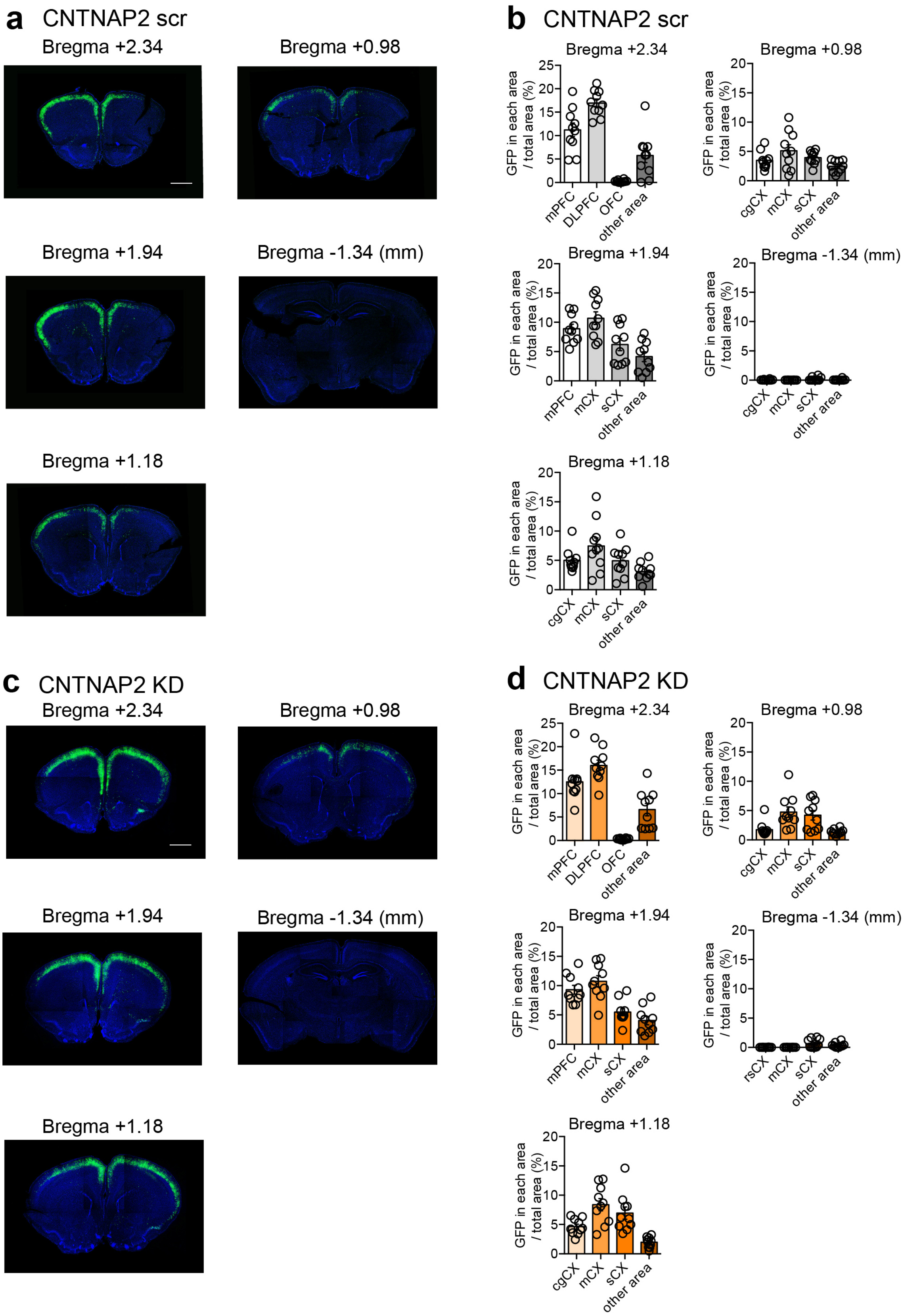

**Supplementary Fig. 3 | Distribution of EGFP expressing transfected cells in the PFC by *in utero* electroporation of CNTNAP2-knockdown or CNTNAP2-scramble vectors.**

**a, c,** Representative rostro-caudal image series of coronal sections of brains (from Bregma: +2.34 mm, +1.94 mm, +1.18 mm, +0.98 mm, and -1.34 mm) from mice transfected CNTNAP2-scramble (CNTNAP2-Scr) (a) and CNTNAP2-knockdown (CNTNAP2-KD) (c) vectors. Blue, staining of nuclei. Scale bar, 1 mm. **b, d** Summary bar graphs showing the distribution of EGFP positive cells in the rostro-caudal series of coronal brain sections from CNTNAP2-Scr (b, n = 10 slices from 10 mice) and CNTNAP2-KD (d, n = 10 slices from 10 mice) mice. Source data are provided as a Source Data file.

**Supplementary Fig. 4**

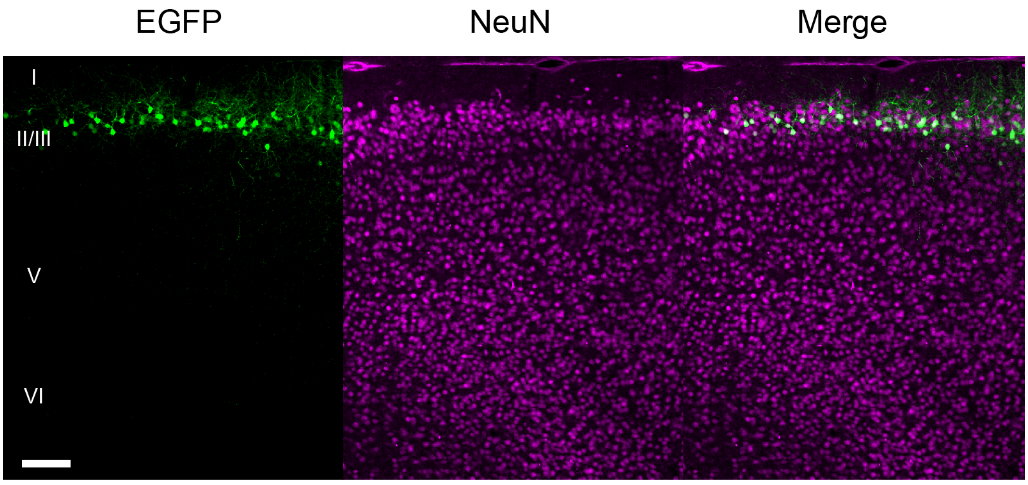


**Supplementary Fig. 4 | Layer specificity in the PFC by *in utero* electroporation.**
Confocal images showing EGFP positive cells (green) and neuronal marker NeuN (magenta) in layer I, II/III, V and VI in the PFC. Scale bar, 100 μm.

**Supplementary Fig. 5**

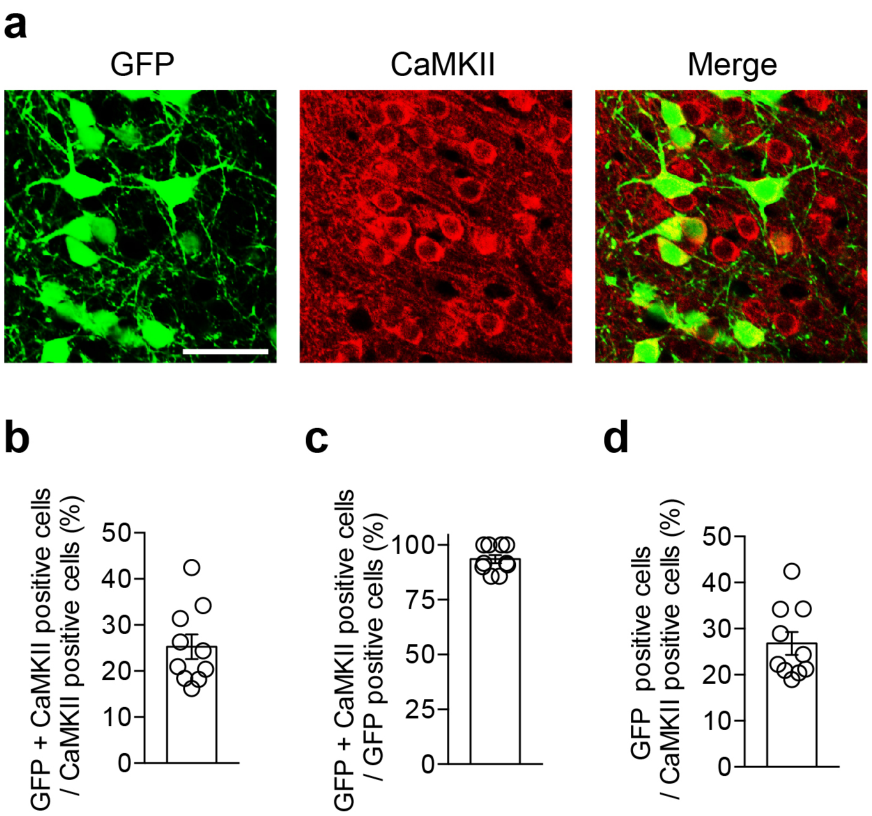


**Supplementary Fig. 5 | Proportion of EGFP expressing transfected cells in CaMKII-positive layer II/III pyramidal neurons of the PFC.**

**a,** Double immunostaining for EGFP (green) and CaMKII (red) in layer II/IIII pyramidal neurons of the PFC. EGFP is expressed preferentially in CaMKII-positive pyramidal neurons. Scale bar, 50 µm. **b-d,** Proportion of EGFP and CaMKII double positive cells to CaMKII positive cells (b), EGFP and CaMKII double positive cells to EGFP positive cells (c), and EGFP positive cells to CaMKII positive cells (d) in CNTNAP2-KD slices (n = 10 images, 5 slices from 3 mice) at postnatal day 21. Source data are provided as a Source Data file.

## Supplementary Fig. 6

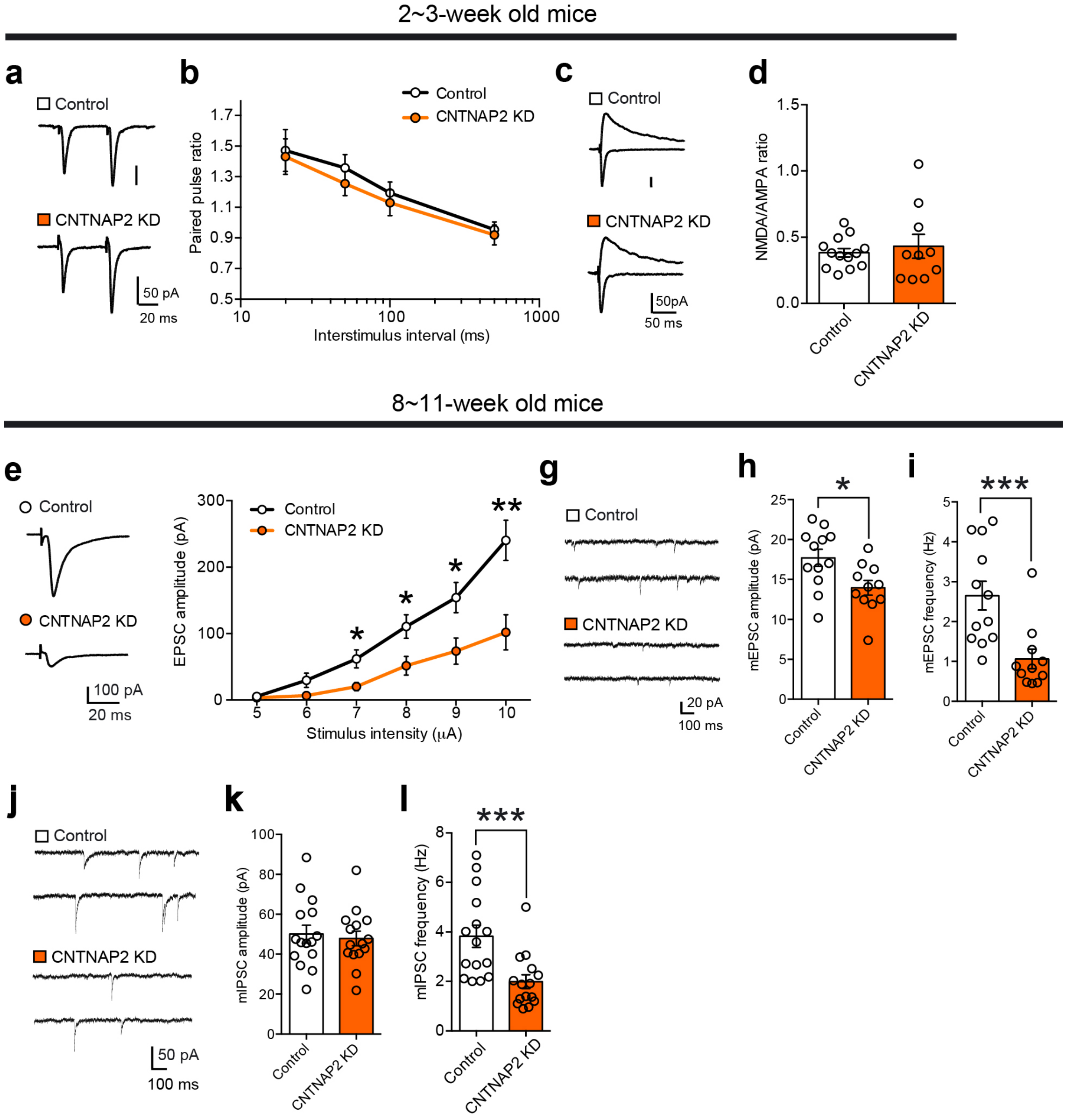

**Supplementary Fig. 6 | Effects of CNTNAP2 knockdown on synaptic function in layer II/III pyramidal neurons of the PFC in young and adult mice.**

**a, b,** Representative traces (a) and average values of paired-pulse ratio (b) for control (white circles, n = 12 cells from 3 mice) and CNTNAP2-KD (orange circles, n = 10 cells from 3 mice) pyramidal neurons in 2-3 week old mice. **c, d,** Representative traces (c) and average values of NMDA/AMPA ratio (d) for control (white column, n = 13 cells from 4 mice) and CNTNAP2-KD, (orange column, n = 10 cells from 4 mice) cells in 2-3 week old mice. **e, f,** Representative traces (e) and averaged input-output relationships (f) for EPSCs induced by stimulation in layer II/III of the mPFC in control (white circles, n = 11 cells from 3 mice) and CNTNAP2-KD (orange circles, n = 10 cells from 3 mice) pyramidal neurons in 8-11 week old mice. The holding potential was -70 mV. **g-i,** Sample traces of mEPSC (g) and summary bar graphs showing the mean amplitude (h) and frequency (i) of mEPSC for control (white columns, n = 12 cells from 4 mice) and CNTNAP2-KD (orange columns, n = 11 cells from 3 mice) cells in 8-11 week old mice. **j-l,** Sample traces of mIPSC (j) and summary bar graphs showing the mean amplitude (k) and frequency (l) of mIPSC for control (white columns, n = 15 cells from 4 mice) and CNTNAP2-KD (orange columns, n = 15 cells from 4 mice) cells in 8-11 week old mice. $^*P < 0.05$, $^{**}P < 0.01$ (Mann-Whitney U test). Source data are provided as a Source Data file.

**Supplementary Fig. 7**

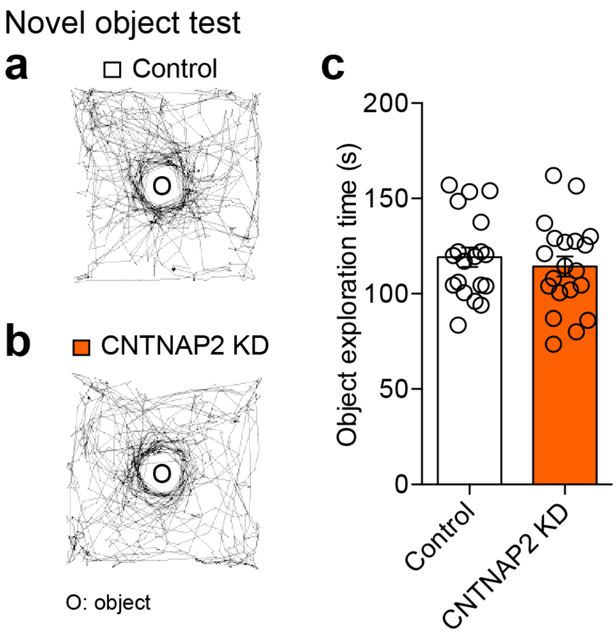


**Supplementary Fig. 7 | Normal exploratory behavior for the novel object in CNTNAP2-knockdown mice.**

**a-c,** Representative tracks (a, b) and summary bar graphs showing the amount of time spent around the object (O) (c) for CNTNAP2-scramble (control) (white columns, n = 19 mice) and CNTNAP2-KD (orange columns, n = 20 mice) mice. Source data are provided as a Source Data file.

**Supplementary Fig. 8**

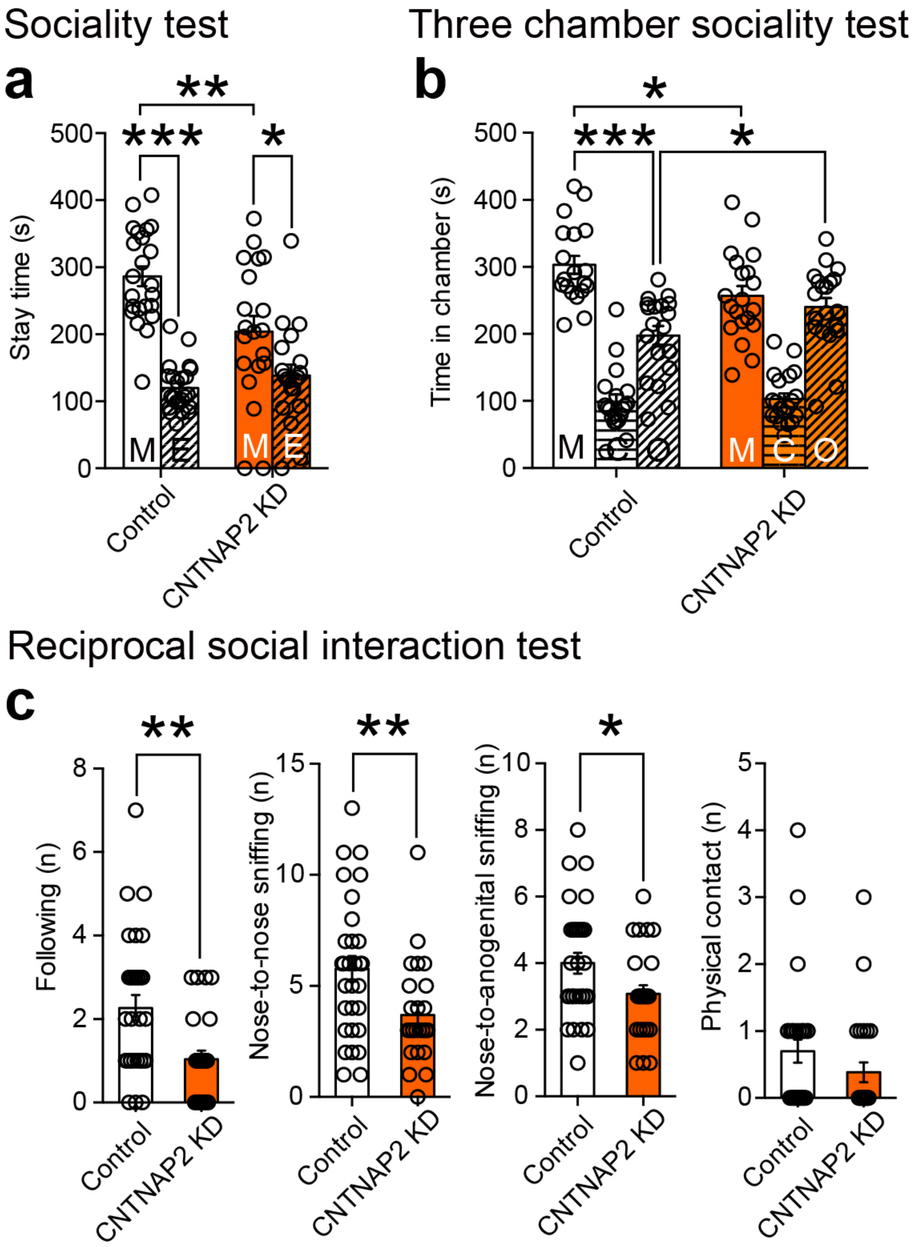

**Supplementary Fig. 8 | Social interaction in CNTNAP2-knockdown mice.**

**a,** Summary bar graphs showing the amount of time spent around the mouse cage (M) and the empty cage (E) in control (white columns, n = 23 mice) and CNTNAP2-KD (orange columns, n = 20 mice) mice. **b,** Summary bar graphs showing the amount of time spent in the mouse chamber (M), the center chamber (C) and the novel object chamber (O) in control (white columns, n = 19 mice) and CNTNAP2-KD (orange columns, n = 20 mice) mice. **c,** Summary bar graphs showing the number of following, nose-to-anogenital sniffing, nose-to-nose sniffing, and physical contact in control (white column, n = 30 mice) and CNTNAP2-KD (orange column, n = 26 mice) mice. *$P$ < 0.05, **$P$ < 0.01, ***$P$ < 0.001 (Student's *t* test or Paired *t* test). Source data are provided as a Source Data file.

**Supplementary Fig. 9**

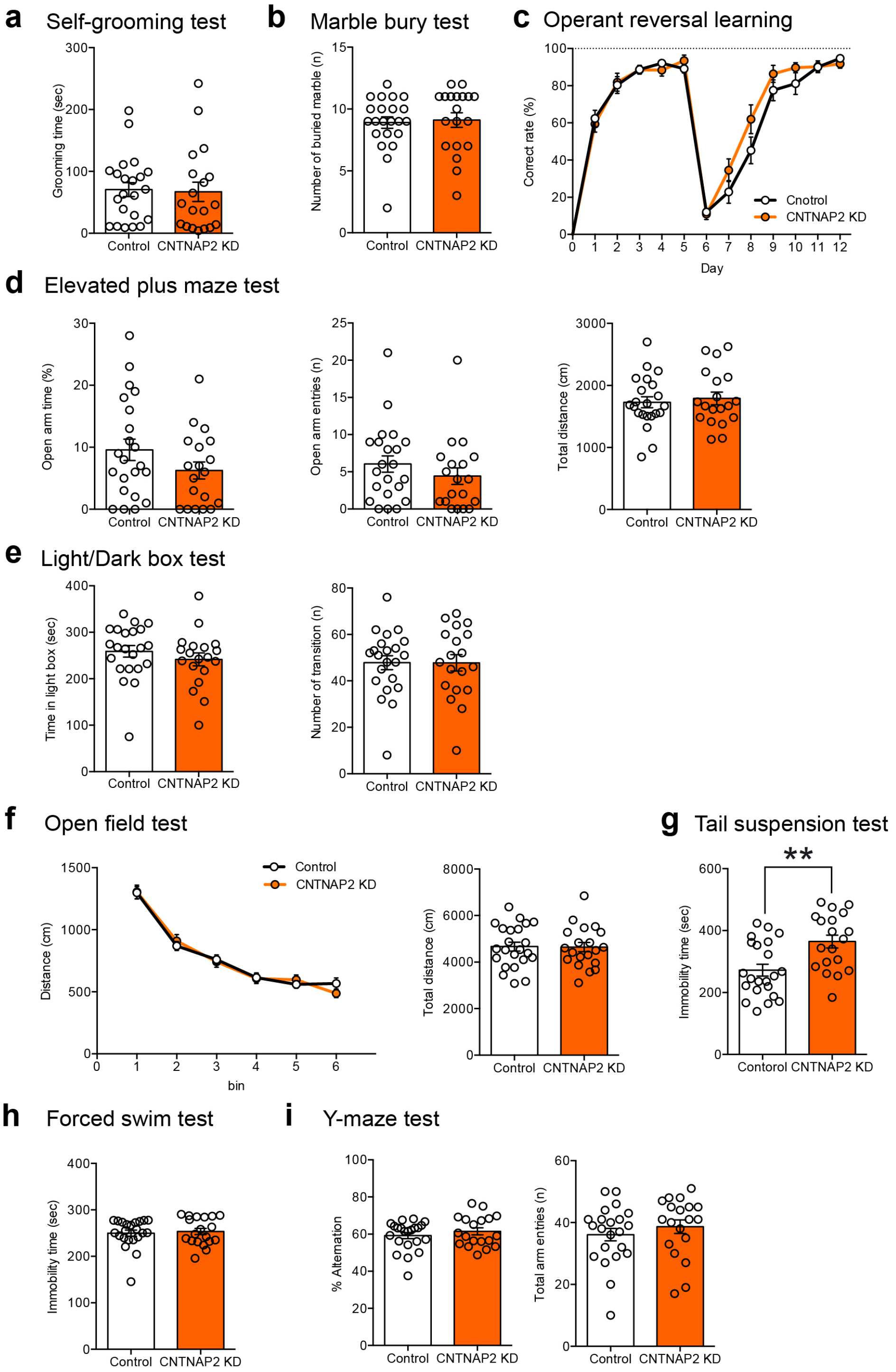

**Supplementary Fig. 9 | Normal repetitive behaviors, behavioral flexibility, anxiety, locomotor activity, and working memory but moderately enhanced depressive behavior in CNTNAP2-knockdown mice.**

**a,** Self-grooming test. Total time spent on grooming in control (white column, n = 22 mice) and CNTNAP2-KD (orange column, n = 19 mice) mice. **b,** Marble bury test. The number of buried marbles in control (white column, n = 22 mice) and CNTNAP2-KD (orange column, n = 19 mice) mice. **c,** Operant reversal learning. Correct rate in control (white column, n = 18 mice) and CNTNAP2-KD (orange column, n = 12 mice) mice. **d,** Elevated plus maze test. Percentage of time in the open arm, number of open arm entries and total distance traveled for control (white column, n = 23 mice) and CNTNAP2-KD (orange column, n = 20 mice) mice. **e,** Light/Dark box test. Time in the light box, latency to the light box entry, and distance traveled for control (white column, n = 22 mice) and CNTNAP2-KD (orange column, n = 19 mice) mice. **f,** Open field test. Total distance traveled shown in 5 min time bins and during 30 min for control (white column, n = 23 mice) and CNTNAP2-KD (orange column, n = 20 mice) mice. **g,** Tail suspension test. Immobility time in control (white column, n = 22 mice) and CNTNAP2-KD (orange column, n = 19 mice) mice. **h,** Forced swim test. Immobility time in control (white column, n = 22 mice) and CNTNAP2-KD (orange column, n = 19 mice) mice. **i,** Y-maze test. Percentage of alternation in control (white column, n = 22 mice) and CNTNAP2-KD (orange column, n = 19 mice) mice. ***$P < 0.01$ (Student's *t* test). Source data are provided as a Source Data file.

**Supplementary Fig. 10**

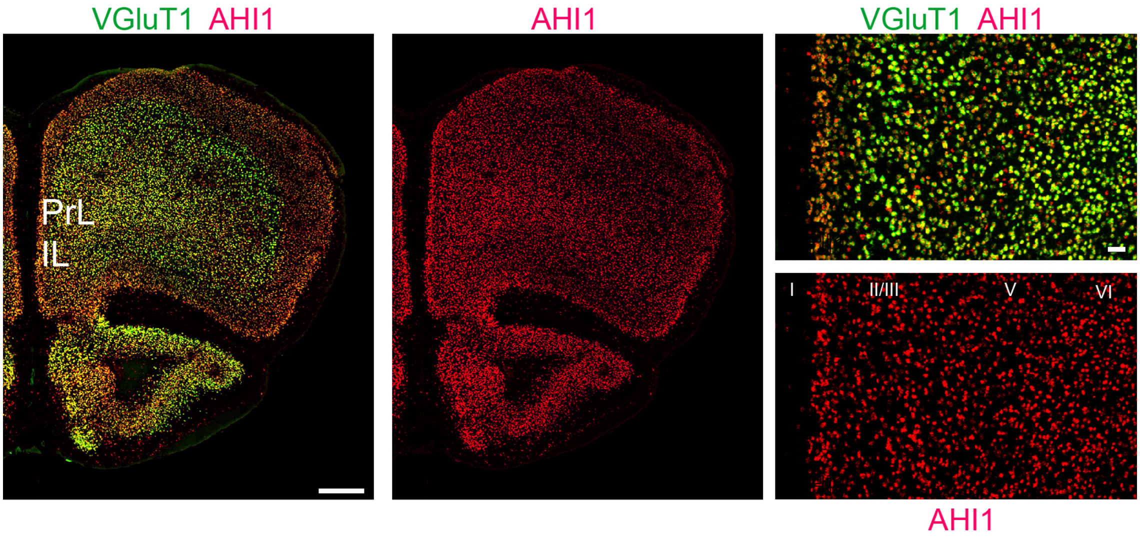


**Supplementary Fig. 10 | Expression of AHI1 in the adult mouse PFC.**

Expression of mRNAs for AHI1 in the adult mouse PFC. Images from double fluorescence in situ hybridization analyses to detect mRNA for AHI1 (red) and VGluT1 (green) in layer I, II/III, V and VI of the PFC. Scale bars, 500 μm (left), 50 μm (right). PrL: pre-limbic, IL: infra-limbic.

**Supplementary Fig. 11**

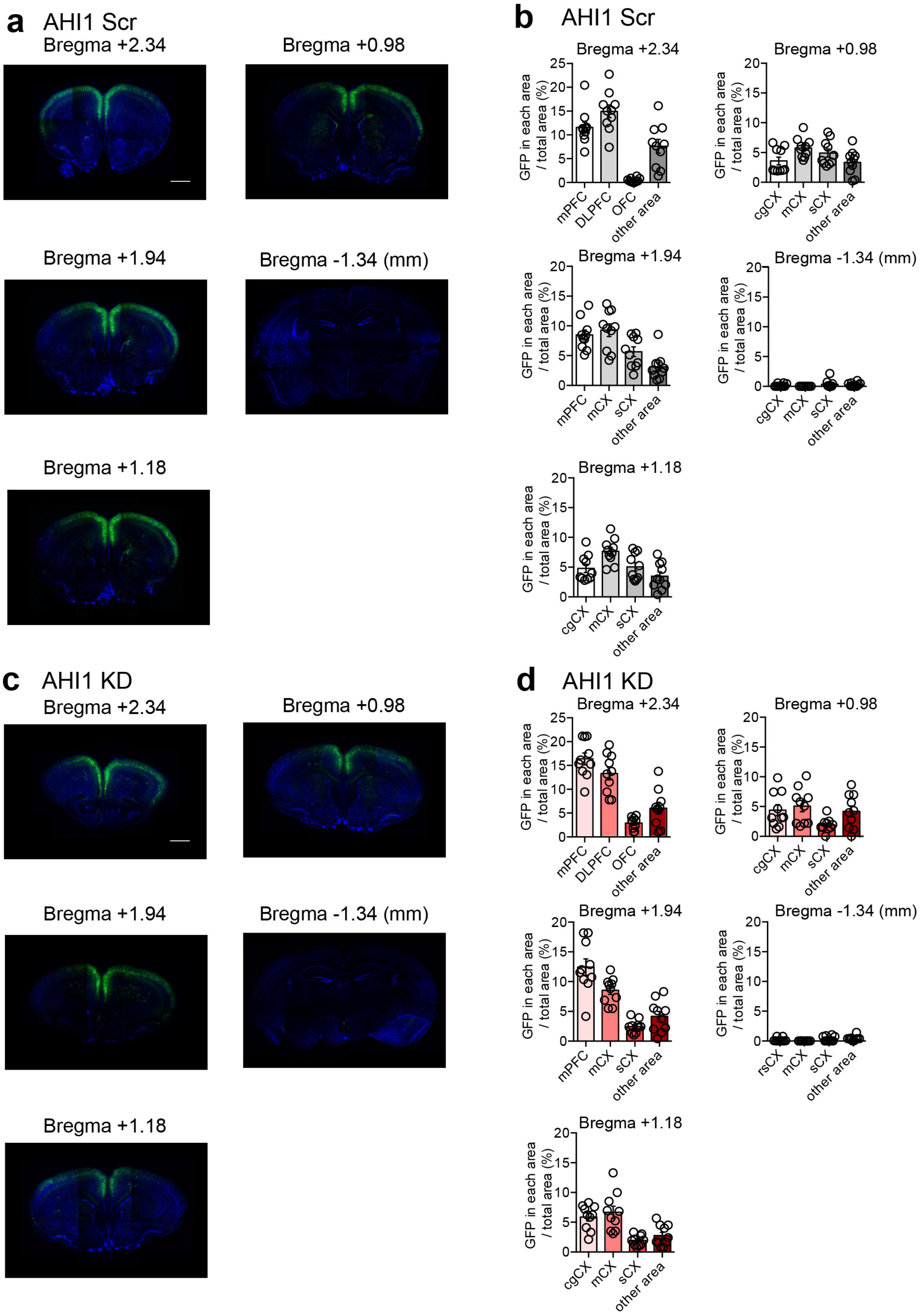

**Supplementary Fig. 11 | Distribution of EGFP expressing transfected cells in the PFC by *in utero* electroporation of AHI1-knockdown or AHI1-scramble vectors.**

**a, c,** Representative rostro-caudal image series of coronal sections of brains (from Bregma: +2.34 mm, +1.94 mm, +1.18 mm, +0.98 mm, and -1.34 mm) from mice transfected AHI1-scramble (AHI1 Scr) **(a)** and AHI1-knockdown (AHI1 KD) **(c)** vectors. Blue, staining of nuclei. Scale bar, 1 mm. **b, d,** Summary bar graphs showing the distribution of EGFP positive cells in the rostro-caudal series of coronal brain sections of AHI1-Scr (b, n = 10 slices from 10 mice) and AHI1-KD (d, n = 10 slices from 10 mice) mice. Source data are provided as a Source Data file.

**Supplementary Fig. 12**

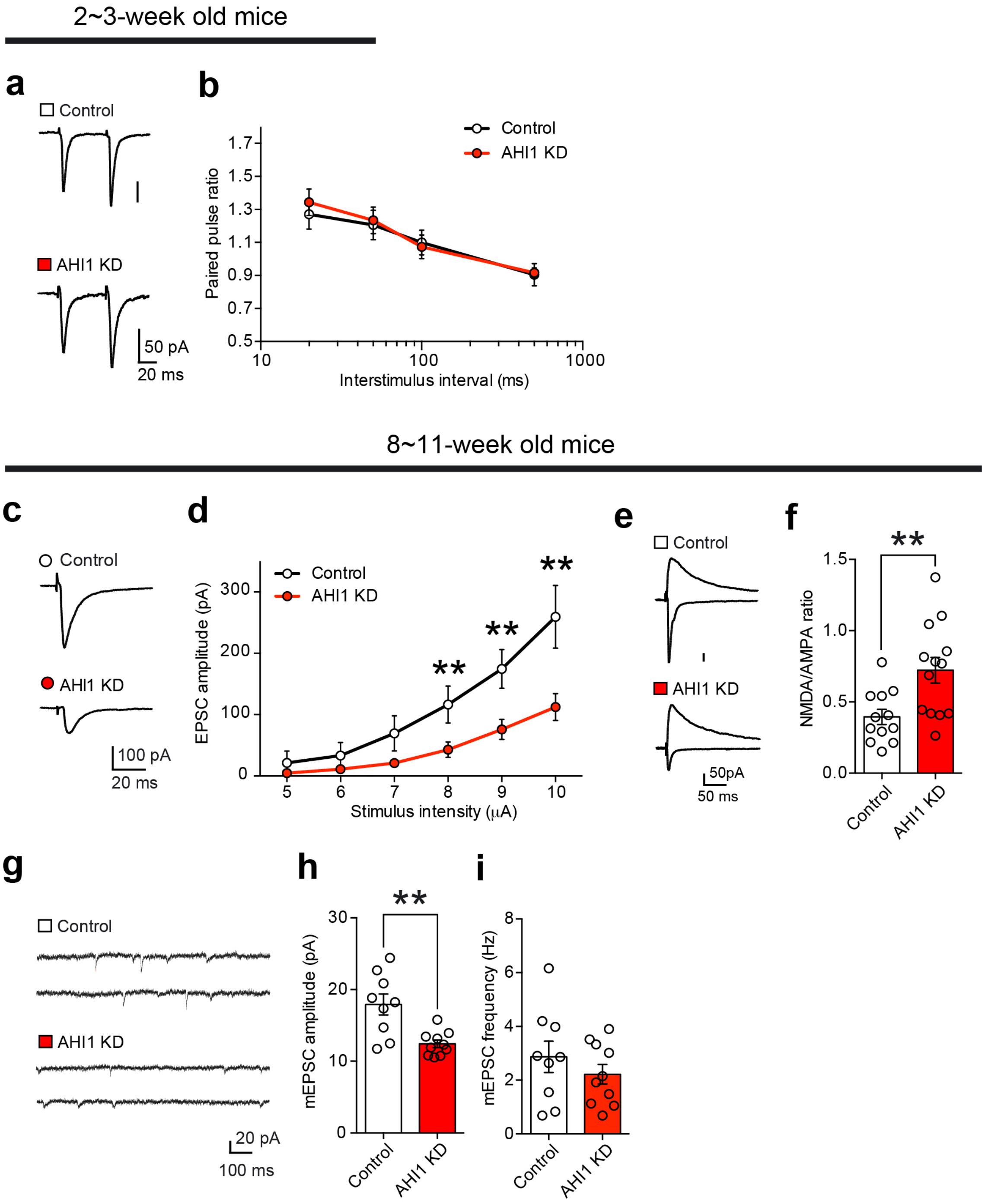

**Supplementary Fig. 12 | Effects of AHI1 knockdown on synaptic function in layer II/III pyramidal neurons of the PFC in young and adult mice.**

**a, b,** Representative traces (a) and average values of paired-pulse ratio (b) for control (white circles, n = 9 cells from 2 mice) and AHI1-KD (red circles, n = 10 cells from 2 mice) pyramidal neurons in 2-3 week old mice. **c, d,** Representative traces (c) and averaged input-output relationships (d) for EPSCs induced by stimulation in layer II/III of the mPFC in control (white circles, n = 16 cells from 6 mice) and AHI1-KD (red circles, n = 19 cells from 6 mice) pyramidal neurons in 8-11 week old mice. The holding potential was -70 mV. **e, f,** Representative traces (e) and average values of NMDA/AMPA ratio (f) for control (white column, n = 12 cells from 5 mice) and AHI1-KD (red column, n = 13 cells from 6 mice) cells in 8-11 week old mice. **g-i,** Sample traces of mEPSC (g) and summary bar graphs showing the mean amplitude (h) and frequency (i) of mEPSC for control (white columns, n = 9 cells from 4 mice) and AHI1-KD (red columns, n = 10 cells from 4 mice) cells in 8-11 week old mice. **$P < 0.01$ (Mann-Whitney U test). Source data are provided as a Source Data file.

# Supplementary Fig. 13

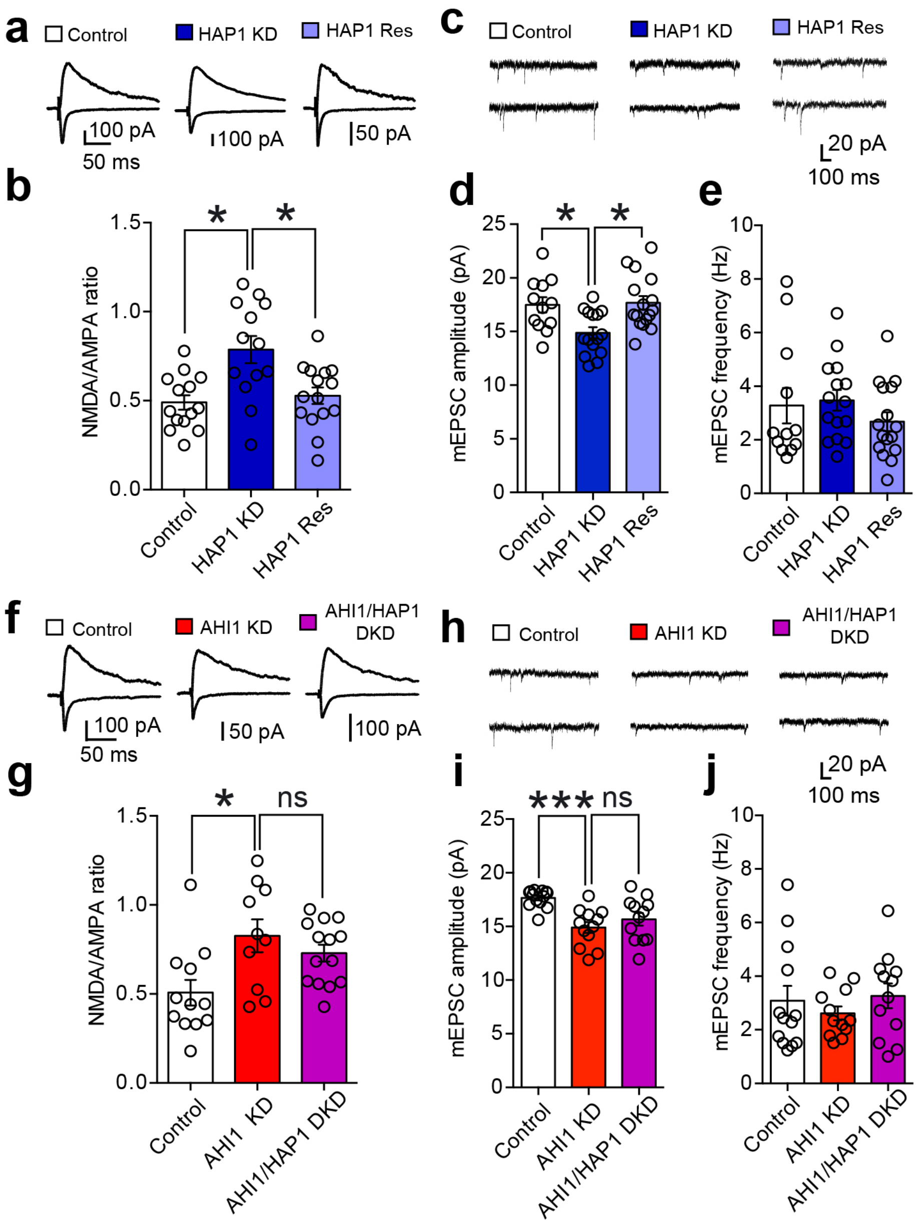

**Supplementary Fig. 13 | Double knockdown of AHI1 and HAP1 exerted the same effects as single knockdown of AHI1 on the NMDA/AMPA ratio and mEPSCs.**

**a, b,** Representative traces (a) and average values of NMDA/AMPA ratio (b) for control (white column, n = 14 cells from 4 mice), HAP1-KD, (blue column, n = 13 cells from 4 mice) and HAP1-Res (light blue column, n = 15 cells from 3 mice) pyramidal neurons. **c-e,** Sample traces of mEPSC (c) and summary bar graphs showing the mean amplitude (d) and frequency (e) of mEPSC for control (white columns, n = 12 cells from 3 mice), HAP1-KD (blue columns, n = 15 cells from 3 mice) and HAP1-Res (light blue columns, n = 16 cells from 3 mice) pyramidal neurons. **f, g,** Representative traces (f) and average values of NMDA/AMPA ratio (g) for control (white column, n = 12 cells from 4 mice), AHI1-KD, (red column, n = 10 cells from 4 mice) and AHI1/HAP1-DKD (purple column, n = 14 cells from 3 mice) pyramidal neurons. **h-j,** Sample traces of mEPSC (h) and summary bar graphs showing the mean amplitude (i) and frequency (j) of mEPSC for control (white columns, n = 13 cells from 3 mice), AHI1-KD (red columns, n = 12 cells from 3 mice) and AHI1/HAP1-DKD (purple columns, n = 12 cells from 3 mice) pyramidal neurons. $^{*}P < 0.05$, $^{***}P < 0.001$ (Dunn test). Source data are provided as a Source Data file.

**Supplementary Fig. 14**

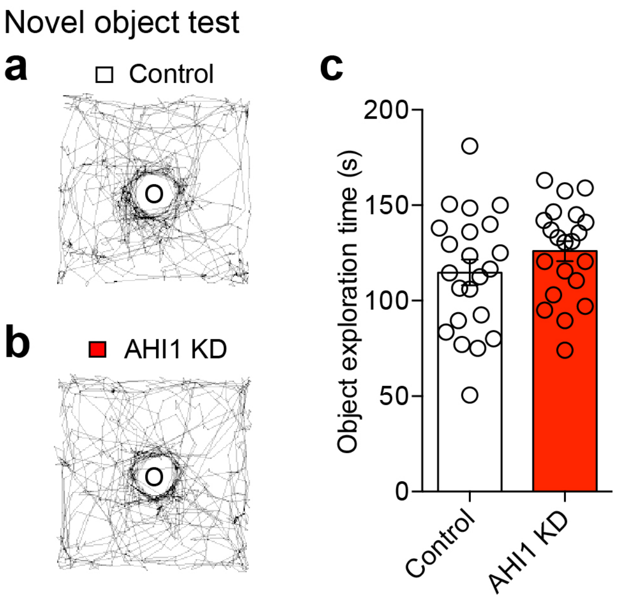


**Supplementary Fig. 14 | Normal exploratory behavior for the novel object in AHI1-knockdown mice.**

**a-c,** Representative tracks (a, b) and summary bar graphs showing the amount of time spent around the object (O) (c) for AHI1-scramble (control) (white columns, n = 22 mice) and AHI1-KD (red columns, n = 21 mice) mice. Source data are provided as a Source Data file.

**Supplementary Fig. 15**

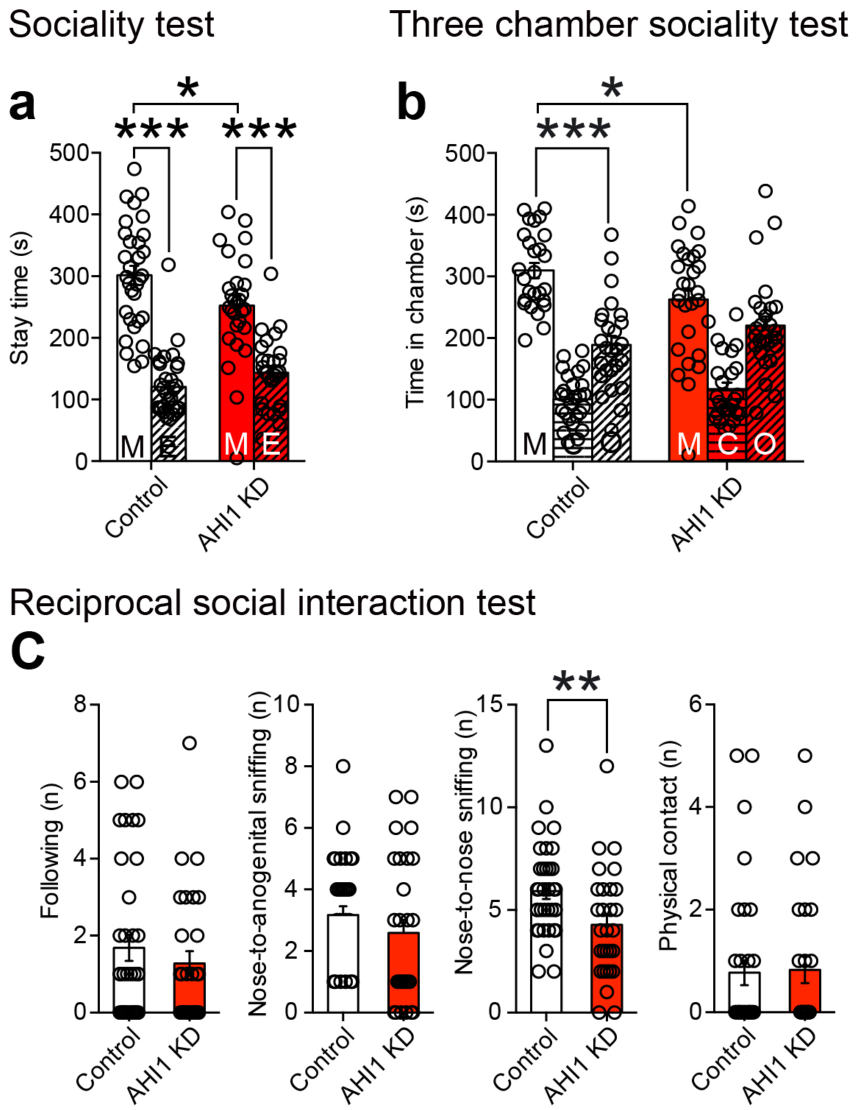

**Supplementary Fig. 15 | Social interaction in AHI1-knockdown mice.**

**a,** Summary bar graphs showing the amount of time spent around the mouse cage (M) and the empty cage (E) in control (white columns, n = 31 mice) and AHI1-KD (red columns, n = 28 mice) mice. **b,** Summary bar graphs showing the amount of time spent in the mouse (M) chamber, the center chamber (C) and the novel object chamber (O) in control (white columns, n = 26 mice) and AHI1-KD (red columns, n = 27 mice) mice. **b,** Summary bar graphs showing the number of following, nose-to-anogenital sniffing, nose-to-nose sniffing, and physical contact in control (white column, n = 35 mice) and AHI1-KD (red column, n = 29 mice) mice. *$P < 0.05$, **$P < 0.01$, ***$P < 0.001$ (Student's *t* test or Paired *t* test). Source data are provided as a Source Data file.

## Supplementary Fig. 16

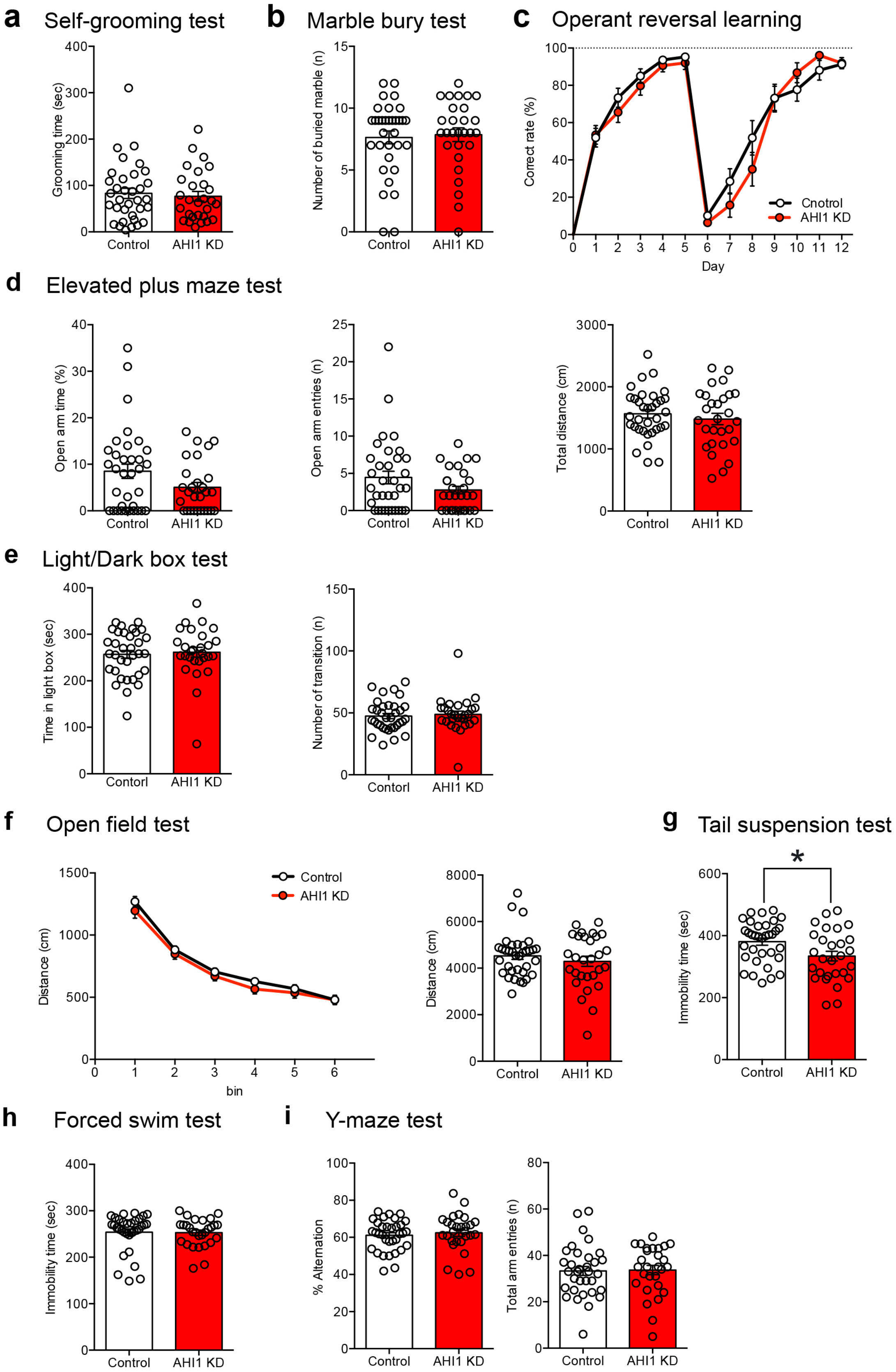

**Supplementary Fig. 16 | Normal repetitive behaviors, behavioral flexibility, anxiety, locomotor activity, and working memory but moderately reduced depressive behavior in AHI1-knockdown mice.**

**a,** Self-grooming test. Total time spent on grooming in control (white column, n = 33 mice) and AHI1-KD (red column, n = 28 mice) mice. **b,** Marble bury test. The number of buried marbles in control (white column, n = 33 mice) and AHI1-KD (red column, n = 28 mice) mice. **c,** Operant reversal learning. Correct rate in control (white column, n = 16 mice) and AHI1-KD (red column, n = 13 mice) mice. **d,** Elevated plus maze test. Percentage of time in the open arm, number of open arm entries and total distance traveled in control (white column, n = 34 mice) and AHI1-KD (red column, n = 28 mice) mice. **e,** Light/Dark box test. Time in the light box, latency to the light box entry, and distance traveled in control (white column, n = 34 mice) and AHI1-KD (red column, n = 28 mice) mice. **f,** Open field test. Total distance traveled shown in 5 min time bins and during 30 min in control (white column, n = 35 mice) and AHI1-KD (red column, n = 29 mice) mice. **g,** Tail suspension test. Immobility time in control (white column, n = 34 mice) and AHI1-KD (red column, n = 28 mice) mice. **h,** Forced swim test. Immobility time in control (white column, n = 34 mice) and AHI1-KD (red column, n = 28 mice) mice. **i,** Y-maze test. Percentage of alternation in control (white column, n = 33 mice) and AHI1-KD (red column, n = 28 mice) mice. *$P < 0.05$ (Student's *t* test). Source data are provided as a Source Data file.

**Supplementary Fig. 17**

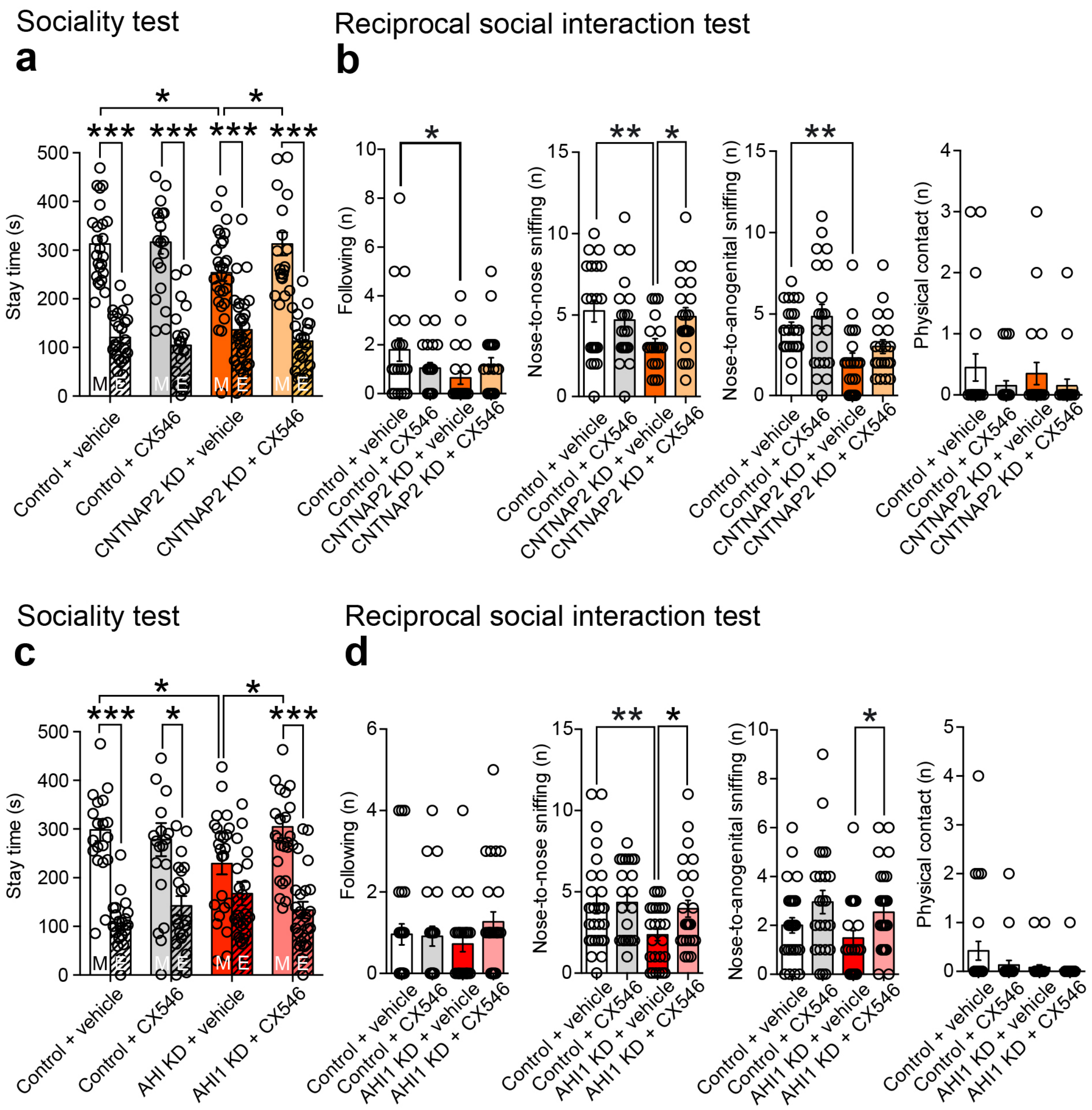

**Supplementary Fig. 17 | Social interaction in CNTNAP2-knockdown and AHI1-knockdown mice after CX546 administration.**

**a,** Summary bar graphs showing the amount of time spent around the mouse cage (M) and the empty cage (E) in vehicle-treated control (control + vehicle) (white column, n = 24 mice), CX546-treated control (control + CX546) (gray column, n = 20 mice), vehicle-treated CNTNAP2-KD (CNTNAP2 KD + vehicle) (orange column, n = 28 mice) and CX546-treated CNTNAP2-KD (CNTNAP2 KD + CX546) (light orange column, n = 20 mice) mice. **b,** Summary bar graphs showing the number of following, nose-to-anogenital sniffing, nose-to-nose sniffing, and physical contact in vehicle-treated control (control + vehicle) (white column, n = 20 mice), CX546-treated control (control + CX546) (gray column, n = 20 mice), vehicle-treated CNTNAP2-KD (CNTNAP2 KD + vehicle) (orange column, n = 20 mice) and CX546-treated CNTNAP2-KD (CNTNAP2 KD + CX546) (light orange column, n = 20 mice) mice. **c,** Summary bar graphs showing the amount of time spent around the mouse cage (M) and the empty cage (E) in vehicle-treated control (control + vehicle) (white column, n = 20 mice), CX546-treated control (control + CX546) (gray column, n = 20 mice), vehicle-treated AHI1-KD (AHI1 KD + vehicle) (red column, n = 24 mice) and CX546-treated AHI1-KD (AHI1 KD + CX546) (light red column, n = 25 mice) mice. **d,** Summary bar graphs showing the number of following, nose-to-anogenital sniffing, nose-to-nose sniffing, and physical contact in vehicle-treated control (control + vehicle) (white column, n = 26 mice), CX546-treated control (control + CX546) (gray column, n = 23 mice), vehicle-treated AHI1-KD (AHI1 KD + vehicle) (red column, n = 25 mice) and CX546-treated AHI1-KD (AHI1 KD + CX546) (light red column, n = 26 mice) mice. $^*P < 0.05$, $^{**}P < 0.01$, $^{***}P < 0.001$ (Student's *t* test or Paired *t* test). Source data are provided as a Source Data file.

# Supplementary Table

| Primer name | | Primer sequence |
|---|---|---|
| CNTNAP2 microRNA1 | Top | 5’-TGCTGTGATCTAGGTGCCAAGGGTCAGTTTTGGCCACTGACTGACTGACCCTTCACCTAGATCA-3’ |
| | Bottom | 5’-CCTGTGATCTAGGTGAAGGGTCAGTCAGTCAGTGGCCAAAACTGACCCTTGGCACCTAGATCAC-3’ |
| CNTNAP2 microRNA2 | Top | 5’-TGCTGTACAAGGTCAATCTCCACATTGTTTTGGCCACTGACTGACAATGTGGATTGACCTTGTA-3’ |
| | Bottom | 5’-CCTGTACAAGGTCAATCCACATTGTCAGTCAGTGGCCAAAACAATGTGGAGATTGACCTTGTAC-3’ |
| CNTNAP2 microRNA3 | Top | 5’-TGCTGATAAGAAGCCAGCATCCTTCCGTTTTGGCCACTGACTGACGGAAGGATTGGCTTCTTAT-3’ |
| | Bottom | 5’-CCTGATAAGAAGCCAATCCTTCCGTCAGTCAGTGGCCAAAACGGAAGGATGCTGGCTTCTTATC-3’ |
| CNTNAP2 microRNA4 | Top | 5’-TGCTGTAGTGCTGAAGCTAAAGTGGAGTTTTGGCCACTGACTGACTCCACTTTCTTCAGCACTA-3’ |
| | Bottom | 5’-CCTGTAGTGCTGAAGAAAGTGGAGTCAGTCAGTGGCCAAAACTCCACTTTAGCTTCAGCACTAC-3’ |
| AHI1 microRNA1 | Top | 5’-TGCTGATAAGATGAAACAGGACGTTCGTTTTGGCCACTGACTGACGAACGTCCTTTCATCTTAT-3’ |
| | Bottom | 5’-CCTGATAAGATGAAAGGACGTTCGTCAGTCAGTGGCCAAAACGAACGTCCTGTTTCATCTTATC-3’ |
| AHI1 microRNA2 | Top | 5’-TGCTGTTGAATGGCAGGTCAGAGTACGTTTTGGCCACTGACTGACGTACTCTGCTGCCATTCAA-3’ |
| | Bottom | 5’-CCTGTTGAATGGCAGCAGAGTACGTCAGTCAGTGGCCAAAACGTACTCTGACCTGCCATTCAAC-3’ |
| AHI1 microRNA3 | Top | 5’-TGCTGAACAGATGCGTACTCGAAGCTGTTTTGGCCACTGACTGACAGCTTCGAACGCATCTGTT-3’ |
| | Bottom | 5’-CCTGAACAGATGCGTTCGAAGCTGTCAGTCAGTGGCCAAAACAGCTTCGAGTACGCATCTGTTC-3’ |
| HAP1 microRNA1 | Top | 5’-TGCTGAACACCAGCACTTCCGATAGCGTTTTGGCCACTGACTGACGCTATCGGGTGCTGGTGTT-3’ |
| | Bottom | 5’-CCTGAACACCAGCACCCGATAGCGTCAGTCAGTGGCCAAAACGCTATCGGAAGTGCTGGTGTTC-3’ |
| HAP1 microRNA2 | Top | 5’-TGCTGATCTGCTGCAGTTTCTCTGTCGTTTTGGCCACTGACTGACGACAGAGACTGCAGCAGAT-3’ |
| | Bottom | 5’-CCTGATCTGCTGCAGTCTCTGTCGTCAGTCAGTGGCCAAAACGACAGAGAAACTGCAGCAGATC-3’ |
| CNTNAP2 Scr1 | Top | 5’-TGCTGACAGTACACGCTGGTTGTGGAGTTTTGGCCACTGACTGACTCCACAACGCGTGTACTGT-3’ |
| | Bottom | 5’-CCTGACAGTACACGCGTTGTGGAGTCAGTCAGTGGCCAAAACTCCACAACGGGCGTGTACTGTC-3’ |
| CNTNAP2 Scr2 | Top | 5’-TGCTGGTAGTCATCAAACCTTATACCGTTTTGGCCACTGACTGACGGTATAAGTTGATGACTAC-3’ |
| | Bottom | 5’-CCTGGTAGTCATCAACTTATACCGTCAGTCAGTGGCCAAAACGGTATAAGGTTTGATGACTACC-3’ |
| CNTNAP2 Scr3 | Top | 5’-TGCTGATGCGCCAACATCCCGAATATGTTTTGGCCACTGACTGACATATTCGGTGTTGGCGCAT-3’ |
| | Bottom | 5’-CCTGATGCGCCAACACCGAATATGTCAGTCAGTGGCCAAAACATATTCGGGATGTTGGCGCATC-3’ |
| CNTNAP2 Scr4 | Top | 5’-TGCTGTGGGGATCAAGATAGTCAGATGTTTTGGCCACTGACTGACATCTGACTCTTGATCCCCA-3’ |
| | Bottom | 5’-CCTGTGGGGATCAAGAGTCAGATGTCAGTCAGTGGCCAAAACATCTGACTAGCTTGATCCCCAC-3’ |
| AHI1 Scr1 | Top | 5’-TGCTGTGGAGGAGTATACACCAAAATGTTTTGGCCACTGACTGACATTTTGGTGTACTCCTCCA-3’ |
| | Bottom | 5’-CCTGTGGAGGAGTACACCAAAATGTCAGTCAGTGGCCAAAACATTTTGGTGTATACTCCTCCAC-3’ |
| AHI1 Scr2 | Top | 5’-TGCTGTTGGCGATAGTACAGTAGGACGTTTTGGCCACTGACTGACGTCCTACTGCTATCGCCAA-3’ |
| | Bottom | 5’-CCTGTTGGCGATAGCAGTAGGACGTCAGTCAGTGGCCAAAACGTCCTACTGTACTATCGCCAAC-3’ |
| AHI1 Scr3 | Top | 5’-TGCTGAACAGCGCGAACTCTAGTGATGTTTTGGCCACTGACTGACATCACTAGATCGCGCTGTT-3’ |
| | Bottom | 5’-CCTGAACAGCGCGATCTAGTGATGTCAGTCAGTGGCCAAAACATCACTAGAGTTCGCGCTGTTC-3’ |
| CNTNAP2 cDNA | Forward | 5’-AGCGAGCTTTTGGAGTACCA-3’ |
| | Reverse | 5’-CCCCAGCTTGTGCTGATAGT-3’ |
| AHI1 cDNA | Forward | 5’-GCAGCACGTGAAGATTCAGA-3’ |
| | Reverse | 5’-CCAAAGGCCACAGTGATTTT-3’ |
| HAP1 cDNA | Forward | 5’-GCTCCGAGCACAGGTTATAAAG-3’ |
| | Reverse | 5’-AGGCAGCAGTTCTAGCCAAG-3’ |

# Supplementary Table | PCR primer sequences.
