# Supplementary material for: Autism spectrum disorder-like behavior caused by reduced excitatory synaptic transmission in pyramidal neurons of mouse prefrontal cortex
Source: Nat Commun. 2020 Oct 12;11:5140. doi: 10.1038/s41467-020-18861-3 (PMC7552417; doi:10.1038/s41467-020-18861-3)
Supplement: Supplementary file 3 — Reporting Summary [file 41467_2020_18861_MOESM3_ESM.pdf]

nature research

Corresponding author(s): Masanobu Kano

Last updated by author(s): Jul 21, 2020

Reporting Summary

Nature Research wishes to improve the reproducibility of the work that we publish. This form provides structure for consistency and transparency in reporting. For further information on Nature Research policies, see Authors & Referees and the Editorial Policy Checklist .

Statistics

For all statistical analyses, confirm that the following items are present in the figure legend, table legend, main text, or Methods section.

n/a	Confirmed	
☐	☒	The exact sample size (*n*) for each experimental group/condition, given as a discrete number and unit of measurement
☐	☒	A statement on whether measurements were taken from distinct samples or whether the same sample was measured repeatedly
☐	☒	The statistical test(s) used AND whether they are one- or two-sided<br>*Only common tests should be described solely by name; describe more complex techniques in the Methods section.*
☒	☐	A description of all covariates tested
☐	☒	A description of any assumptions or corrections, such as tests of normality and adjustment for multiple comparisons
☐	☒	A full description of the statistical parameters including central tendency (e.g. means) or other basic estimates (e.g. regression coefficient) AND variation (e.g. standard deviation) or associated estimates of uncertainty (e.g. confidence intervals)
☐	☒	For null hypothesis testing, the test statistic (e.g. *F*, *t*, *r*) with confidence intervals, effect sizes, degrees of freedom and *P* value noted<br>*Give P values as exact values whenever suitable.*
☒	☐	For Bayesian analysis, information on the choice of priors and Markov chain Monte Carlo settings
☒	☐	For hierarchical and complex designs, identification of the appropriate level for tests and full reporting of outcomes
☒	☐	Estimates of effect sizes (e.g. Cohen's *d*, Pearson's *r*), indicating how they were calculated

Our web collection on statistics for biologists contains articles on many of the points above.

Software and code

Policy information about availability of computer code

Data collection	Electrophysiology, immunohistochemistry or behavioral data were acquired using the patchmaster software (HEKA Elektronik), a FV1200 confocal laser scanning microscope (Olympus), the imageJ software (NIH), the TimeOF4 software (O'Hara & Co.), the TimeEP1 software (O'Hara & Co.), the TimeOF4 software (O'Hara & Co.), the TimeLD4 software (O'Hara & Co.), the TimeFZ2 software (O'Hara & Co.), the TimeYM1 software (O'Hara & Co.), the ultrasonic microphone UltraSoundGate CM 16 (Avisoft Bioacoustics) and an Avisoft recorder (version 4.2.16; Avisoft Bioacoustics).
Data analysis	ImageJ (FIJI version 1.52q) Graphpad Prism6 Software (San Diego, CA, USA).TimeOF4 software (O'Hara & Co.),

For manuscripts utilizing custom algorithms or software that are central to the research but not yet described in published literature, software must be made available to editors/reviewers. We strongly encourage code deposition in a community repository (e.g. GitHub). See the Nature Research guidelines for submitting code & software for further information.

Data

Policy information about availability of data

All manuscripts must include a data availability statement. This statement should provide the following information, where applicable:

- Accession codes, unique identifiers, or web links for publicly available datasets
- A list of figures that have associated raw data
- A description of any restrictions on data availability

The authors declare that the data supporting the findings of this study are available within the article, its Supplementary Information files, the Source Data file or from the corresponding author upon reasonable request. Source data are provided with this paper.

Field-specific reporting

Please select the one below that is the best fit for your research. If you are not sure, read the appropriate sections before making your selection.

- [x] Life sciences
- [ ] Behavioural & social sciences
- [ ] Ecological, evolutionary & environmental sciences

For a reference copy of the document with all sections, see nature.com/documents/nr-reporting-summary-flat.pdf

Life sciences study design

All studies must disclose on these points even when the disclosure is negative.

Sample size	The sample size was estimated based on previous repoprts. We conducted the experiments using more than 3 samples per group.
Data exclusions	For behavioral experiments, we excluded the data when mouse track was not detected correctly. Electrophysiological experiments were not analysed if a noisy recording condition was present.
Replication	Our research findings are consistent with the results of several previous studies:<br>Effects of CNTNAP2 knockdown on synaptic function (Figure 1).<br>Anderson, G. R. et al. Candidate autism gene screen identifies critical role for cell-adhesion molecule CASPR2 in dendritic arborization and spine development. Proc Natl Acad Sci U S A 109, 18120-18125 (2012)<br>Lazaro, M. T. et al. Reduced Prefrontal Synaptic Connectivity and Disturbed Oscillatory Population Dynamics in the CNTNAP2 Model of Autism. Cell Rep. 27, 2567-2578 (2019)<br>Effects of HAP1 knockdown on synaptic function (Supplementary Figure 8,).<br>Mandal M. et al. Impaired alpha-amino-3-hydroxy-5-methyl-4- isoxazolepropionic acid (AMPA) receptor trafficking and function by mutant huntingtin. J Biol Chem 286, 33719-33728 (2011)<br>Effects of AMPA receptors PAM on social behavior in autism mouse models (Figure 6).<br>Kim JW. et al. Pharmacological modulation of AMPA receptor rescues social impairments in animal models of autism. Neuropsychopharmacology. 44, 314-323 (2019)
Randomization	All mice were allocated into age-matched experimental groups.
Blinding	Blinding was used for most of behavior tests.

Reporting for specific materials, systems and methods

We require information from authors about some types of materials, experimental systems and methods used in many studies. Here, indicate whether each material, system or method listed is relevant to your study. If you are not sure if a list item applies to your research, read the appropriate section before selecting a response.

Materials & experimental systems

n/a	Involved in the study	
[ ]	[x]	Antibodies
[ ]	[x]	Eukaryotic cell lines
[x]	[ ]	Palaeontology
[ ]	[x]	Animals and other organisms
[x]	[ ]	Human research participants
[x]	[ ]	Clinical data

Methods

n/a	Involved in the study	
[x]	[ ]	ChIP-seq
[x]	[ ]	Flow cytometry
[x]	[ ]	MRI-based neuroimaging

Antibodies

Antibodies used	Rat monoclonal anti-GFP(Nacalai Tesque, Cat# 04404-84, RRID: AB_10013361)<br>Mouse monoclonal anti-CaMKII antibody (Abcam,Cat# ab22609, RRID:AB_447192)<br>Mouse Anti-NeuN (Merckmillipore, Cat# MAB377 RRID:AB_2298772)
Validation	All antibodies used in this study have been tested by the company and have been cited by other papers.

Eukaryotic cell lines

Policy information about cell lines

Cell line source(s)	HEK293T cells (ATCC) were obtained.

Authentication	HEK293T cells were not authenticated.
Mycoplasma contamination	We have not examined mycoplasma contamination in HEK293T cells.
Commonly misidentified lines (See ICLAC register)	There were no commonly misidentified cell lines used in all experiments.

Animals and other organisms

Policy information about studies involving animals; ARRIVE guidelines recommended for reporting animal research

Laboratory animals	ICR mice were used in the present study. C57/B6 and DBA2 mice were also used in the social novelty and the reciprocal social interaction test.
Wild animals	Not applicable.
Field-collected samples	Not applicable.
Ethics oversight	All experiments were performed in accordance with the guidelines set down by the experimental animal ethics committees of the University of Tokyo, Hokkaido University and the Japan Neuroscience Society.

Note that full information on the approval of the study protocol must also be provided in the manuscript.
